# Precise Two-Dimensional Tilt Measurement Sensor with Double-Cylindrical Mirror Structure and Modified Mean-Shift Algorithm for a Confocal Microscopy System

**DOI:** 10.3390/s22186794

**Published:** 2022-09-08

**Authors:** Zhiyi Wang, Tingyu Wang, Yongqiang Yang, Yukai Yang, Xiaotao Mi, Jianli Wang

**Affiliations:** 1 Changchun Institute of Optics, Fine Mechanics and Physics, Chinese Academy of Sciences, Changchun 130033, China; 2 College of Materials Science and Opto-Electronic Technology, University of Chinese Academy of Sciences, Beijing 100049, China

**Keywords:** confocal microscopy, optical profilometry, peak-extraction algorithm, partition-fitting strategy, position, two-dimensional tilt

## Abstract

To improve the accuracy of three-dimensional (3D) surface contour measurements of freeform optics, a two-dimensional (2D) tilt measurement sensor for confocal microscopy (CM) systems is proposed based on a double-cylindrical mirror structure. First, the proposed system is accurately modeled. Second, we introduce a modified mean–shift-based peak-extraction algorithm with a novel kernel function (MSN) because the reflectivity of the measured object and fluctuation of the light source affect the measurement accuracy. Third, a partition fitting (PF) strategy is proposed to reduce the fitting error and improve the measurement accuracy. Simulations and experiments reveal that the robustness, speed, and angular prediction accuracy of the system effectively improved as a function of MSN and PF. The developed sensor can measure the 2D tilt, where each tilt is a composition of two separate dimensions, and the mean prediction errors in the 2D plane from −10°–+10° are 0.0134° (0.067% full scale (F.S)) and 0.0142° (0.071% F.S). The sensor enables the optical probe of a traditional CM to obtain accurate and simultaneous estimates of the 2D inclination angle and spatial position coordinates of the measured surface. The proposed sensor has potential in 3D topographic reconstruction and dynamic sampling rate optimization for 3D contour detection.

## 1. Introduction

As an emerging optical technology, freeform optics is revolutionary to the development of optics [1]. The high degree of optical freedom enables it to correct aberrations of spherical and aspherical surfaces, thus enhancing the imaging quality, expanding the field-of-view, and reducing the number and weights of system units [2,3,4]. The rapid development of free-form surfaces introduces new requirements for free-form surface inspection, and the inspection accuracy determines the upper limit of manufacturing accuracy. Currently, to achieve high-precision detection of parameters, such as the shape and surface shape of the processed free-form surface mirror in the ultra-precision manufacturing of optical components, the measurement probes used in the measurement equipment must have a high-spatial resolution at the nanometer level coupled with special technical characteristics, such as noncontact [5]. The probe is driven by the 3D transmission mechanism to scan and sample the 3D surface before the point cloud algorithm restores it.

Owing to its high precision, fast acquisition, real-time visualization, and unique axial tomographic capabilities, confocal microscopy (CM) is extensively utilized in biomedical applications [6,7,8,9], materials science [10,11], optical topography [12], and geometrical measurements of industrial precision parts [13]. The lateral resolution of the confocal microscope is 1.4 times higher than that of ordinary microscopes with the same numerical aperture. The axial resolution of the confocal microscope reaches submicron levels and primarily depends on the width and sampling rate of the measured intensity profile obtained by axially scanning the sample through the focal point of the microscope objective. Therefore, CM depends on the sensitivity of the drive moving the sample. The axial response curve represents the shape of the vertical intensity profile, and if the optical element is free of aberrations, the axial response curve exhibits a ${\sin c}^{2}$-like shape [14].

When measuring the topography of a machined surface, several factors affect classical confocal microscopes, such as poor light source stability, ambient stray light, photodetectors, and common-mode noise of the measurement circuit. The laser differential confocal microscope (LDCM) is based on dividing the measured signal into two channels on the receiving side of the confocal optical path. It differentially connects two photoelectric converters to obtain a confocal differential signal. The limitations of the optical focusing detection method, such as large linear errors and small measurement range, can be overcome by using the differential confocal signal as the measurement signal of the engineered surface. However, the noise of the light source and detector affects the accuracy. Moreover, common-mode noise types, such as the light intensity drift of the light source and electronic drift of the detector, are eliminated, thus considerably improving the signal-to-noise ratio (SNR) of the measurement. The improvement can be attributed to the advantage of LDCM performing measurements over a large range during operation, such as maximum slope signal measurements with the characteristics of both the bipolar detection of the LDCM signal and the existence of an absolute zero at the maximum slope of the measured signal [15,16], which represents the intersection point of two curves. Owing to such advantages, CM and LDCM are extensively utilized in industrial applications, and they are ideal optical probes [17,18,19] for the three-dimensional (3D) contour detection of free-form surfaces [20].

Furthermore, the surface inclination angle of each position is crucial when acquiring the spatial 3D coordinates of each point on tested optical curved surfaces using the optical probe of the confocal structure [21]. Unlike the detection method that only obtains the coordinates of discrete points, simultaneous acquisition has several advantages. First, the ability to detect small defects during 3D reconstruction can be enhanced to improve the accuracy when the position of the measured discrete point and the surface inclination of the discrete point are simultaneously obtained. Second, accurate measurements of surface inclination can be effectively used to predict the location of adjacent points, thus allowing dynamic optimization of scanning strategies and lateral sampling rates. Finally, either CM or LDCM is employed as an optical probe. When the measured surface is tilted, the peak position and absolute zero point do not change without considering the aberration. However, the sensitivity and resolution of the measurement system are different. Compensation is required if the inclination angle exceeds a certain limit. Compared with the detection method that solely obtains the slope angle of the surface to be measured, there is no accumulated error, and each measurement point is independent of the others. Thus, subsequent complex algorithm processing is not required. Notably, both CM and LDCM use an axial scanning mechanism for fast scanning to obtain the energy distribution on the detector. However, this imposes more stringent requirements for the response speed associated with simultaneous angle measurements.

Several studies have attempted to simultaneously measure the position and tilt [21,22,23,24,25,26]. Yang [22] calculated the distance from the sensor to the surface, inclination angle of the surface to be measured, and roughness by using a reflective optical sensor based on intensity modulation to receive different intensity combinations of eight optical fibers. An average ranging accuracy of 15 μm and an angle measurement accuracy of 0.8° were achieved. Admittedly, this method is simple, cheap, and easy to design. However, the accuracy is low, and it cannot meet the system requirements. Neuschaefer–Rube [23] proposed an autofocus system based on reflection ellipsometry, which was successfully applied to perform precise measurements of smooth surfaces. The method has high-lateral resolution and can simultaneously obtain both position and inclination. Simultaneously obtaining the surface inclination angle and spatial coordinates using the confocal structure in the measurement system is of great engineering significance. However, existing publications on the topic are limited. Pribosek [24] utilized a set of optical prisms to encode the illumination light in one dimension, divided the aperture into eight subapertures, decoded the reflected light, and determined the angle by the intensity contribution of each subaperture. However, the theoretical model cannot deal with 2D input intensity distributions and defocusing. More importantly, the confocal system can only measure one-dimensional (1D) tilts, so its application range is limited. Henselmans et al. [25] measured the 2D transverse displacement of the back-reflected beam by adding an additional beam splitter and a 2D tetralateral position-sensing detector (PSD) to the reflected beam path of a differential confocal system. The achieved tilt measurement uncertainty (2*σ*) was 0.11° over a measurement range of −5°–+5°.

Although the angle measurement module yields a very high-response speed after using the PSD, given the influences of the principle and structure of the PSD itself, numerous factors (such as material inhomogeneity, electrode shape, and edge effect) can result in a nearly linear relationship between the estimated and the actual position when the light spot is in the center of the PSD. Consequently, a large nonlinear error is produced when the spot is slightly offset, which requires heavy calibration and algorithmic compensation. Meanwhile, small-sized commercial PSDs have a smaller linear effective area that cannot accommodate system structures using larger beams. Therefore, the method requires a small spot, or is suitable for a spot whose displacements on the PSD are an order of magnitude smaller than the spot diameter. Additionally, the overall energy of the returned beam changes when the confocal system measures an object with uneven reflectivity; this induces a large change in the SNR. The structure using PSD cannot control the stability of accuracy using advanced extraction algorithms, which results in a greater accuracy impact. Conchello [26] proposed a confocal system that placed a rotating aperture in the beam path to estimate the distribution of reflected light on the back focal path, thus enabling surface inclination measurements. In addition, the scheme can record the tilt angle in any direction and can fully utilize the numerical aperture (NA) of the microscope’s objective lens. However, the rotation of the diaphragm can cause unnecessary errors owing to the vibration that affects the measurement accuracy. Wu [27] proposed a novel method to combine diffraction image microscopy (DIM) with artificial neural networks. Accordingly, many diffraction images with different 2D tilt angles were collected to learn the mapping relationship between the diffraction image and the corresponding surface orientation that successfully realized the simultaneous acquisition of angle and position. Furthermore, the repeatability of the obtained surface inclination was 0.037°. Overall, several scholars have attempted to address existing problems, such as small measurement range, low accuracy, low robustness, complex structure, and insufficient data processing speed to satisfy the system requirements. However, a system that can simultaneously obtain the position and tilt is urgently required to optimize the measurement ability of the confocal probe, simultaneously obtain the angle and position, and satisfy the accuracy and response speed requirements of the confocal probe for three-dimensional (3D) measurements. 

In this study, we propose a precise, two-dimensional tilt measurement sensor applicable in CM or LDCM to simultaneously acquire spatial position and 2D tilt. First, we propose a spatially orthogonal double cylindrical mirror structure. The combination of each cylindrical mirror and linear CCD significantly reduces the dark noise inherent to the camera. Thus, we ensure the response speed of the surface array CCD satisfies the system requirements, and it uses an extremely high response speed linear array CCD to enhance the system response of the sensor. The proposed structure successfully reduced a two-dimensional angle measurement into a one-dimensional peak finding problem by effectively utilizing the high-resolution characteristic of linear array CCD, and enabling the use of advanced algorithms in the process of peak extraction to improve the processing speed, precision, and robustness of the sensor. Sensors with a PSD structure cannot achieve this. Second, regarding the mathematical model, this study did not apply the approximation that the return beam is round on the exit pupil surface of the microscope objective lens, which is unique to this study. The mathematical model proposed in this paper considers the influence of the system disturbances that the sensor may encounter in the operating state on the output of the linear CCD, such as speckle, environmental noise, etc. In terms of data processing, a modified mean–shift-based peak-extraction algorithm with a novel kernel function (MSN) is proposed to extract the peak. Moreover, a partition fitting (PF) strategy is proposed for the calibration surface fitting process. The use of MSN balances the system demands in terms of accuracy and processing speed. The robustness and anti-noise ability of the system are improved. Finally, the PF strategy enhances the prediction accuracy of the system.

This remainder of this paper is organized as follows: Section 2 describes the approximate mathematical model describing the light intensity distribution on a line-array CCD during 2D tilting, and the system structure proposed herein is used. The MSN peak-extraction and partition-fitting algorithms are described in Section 3. Simulation results are presented in Section 4. In Section 5, the feasibility of the proposed system and the efficiency of the revised algorithm are verified via physical experiments. In Section 6, we present the conclusions of this study.

## 2. Numerical Model and System Design

**Figure 1:**
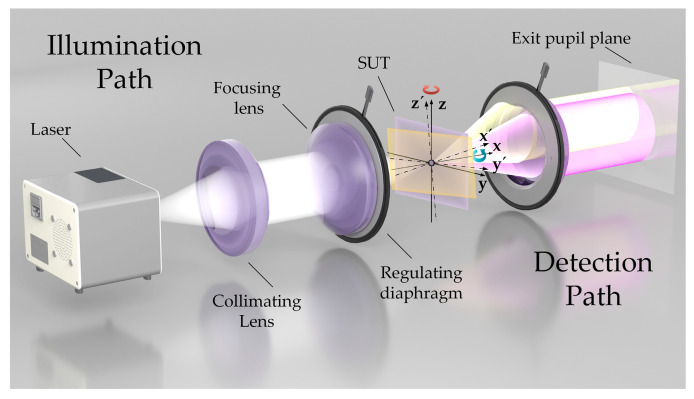
Optical setup of a typical unfolded confocal system.

For illustrative purposes, a reflective laser confocal microscope system is deployed. Figure 1 illustrates the light propagation path of a confocal microscope. Without considering the influence of aberration, the collimated Gaussian beam is focused on the object to be measured after passing through the microscope objective [24]. An aperture that is slightly smaller than the diameter of the microscopic objective lens is placed on the focusing side of the objective to reduce the influences of ambient stray light and sidewall of the microscope objective. The diaphragm is regarded as an ideal pupil plane to simplify the proposed model, and the light field distribution of the plane is identical to that of the exit pupil plane of the microscopic objective lens.

The objective of the microscope emits a cone-shaped illumination light. It forms a diffraction-limited spot on the surface of the measured object placed at the focal length of the microscope objective. The laws of reflection apply to the system when the surface under test (SUT) is a smooth surface within the spot.

**Figure 2:**
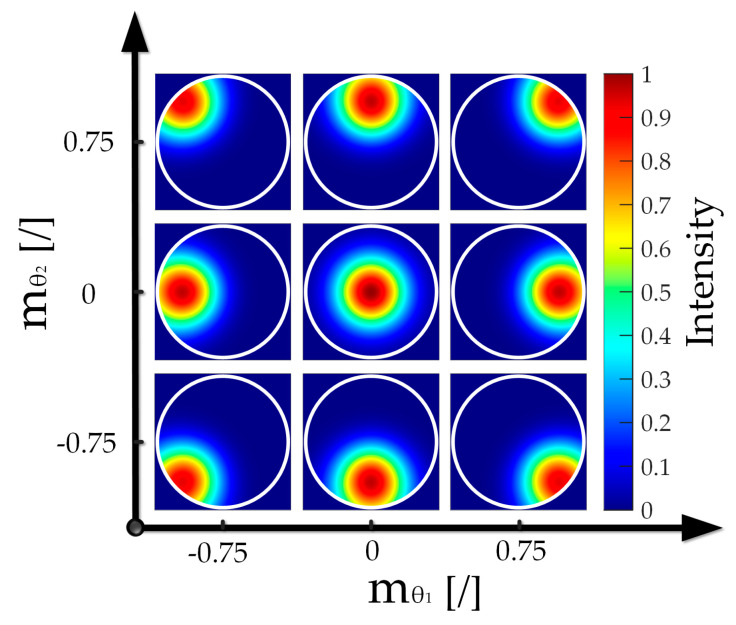
Intensity distribution at the exit pupil plane when tilted in two-dimensions (2D).

If the surface of the measured object is perpendicular to the optical axis, the returned beam is a purple conical beam that appears as a complete circle on the exit pupil’s plane [26], as shown in Figure 1. If the SUT is tilted, the reflected light cone beam tilts twice as much, which induces an offset associated with the returning beam. A part of the area in the pupil’s plane does not receive light, and the diaphragm blocks part of the light in the opposite direction of that area. For the yellow conical beam displayed in Figure 1, the intensity distributions of the light field on the pupil’s plane of the microscope objective are shown in Figure 2. Owing to the difficulty in uniformly measuring the tilt angle in different systems, the axis of the tilted return beam of any dimension is defined at the focal point of the diaphragmatic plane. Thus, the ratio $m_{\theta }$ of the distance ftan2θ from the intersection of the diaphragm plane to the incident optical axis and the radius *R* of the diaphragm is defined as the relative inclination angle [27], where *f* indicates the focal length of the microscope’s objective and *θ* represents the tilt angle.

**Figure 3:**
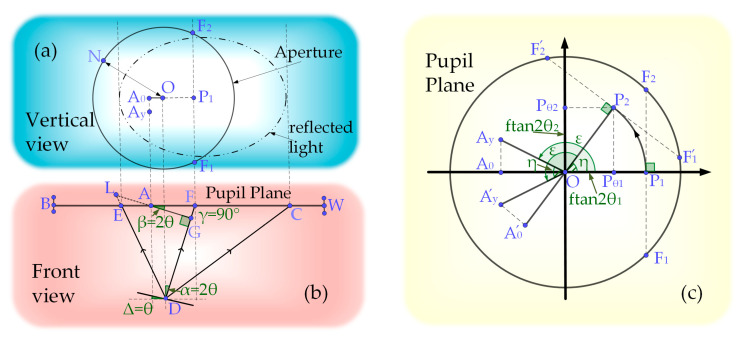
Ray tracing of the returning beam on the object side of the microscope during a 1D tilt illustrating (**a**) the top and (**b**) front views; (**c**) conversion relationship between 1D and two-position tilts.

A simple theoretical model is proposed to study the confocal response as a function of the surface tilt. The developed model predicts the relationship between the light intensity distribution at the exit pupil plane and the 2D tilt. To derive this relation, the proposed model considers two conditions. First, the object surface was placed exactly at the front focal plane of the microscope’s objective lens. Second, the objective was considered as an adaxial surface. The first step involved studying the optical system, and an analysis of the top and front views of the focusing system indicates that 1D tilt occurs, as shown in Figure 3a. Based on the first step, we analyzed the 2D tilt, as shown in Figure 3b.

The radius of the incident parallel Gaussian beam is *r*, which represents the distance between the position where the maximum amplitude drops to a position with a maximum amplitude of 1e2. In the top view, the dotted eccentric ellipse represents the spot profile of the returning conical beam and surface of the diaphragm. The solid line circle represents the profile of the diaphragm with radius *R*. *O*(0, 0) represents the center of the optical axis, *I_0_* represents the center intensity of the incident beam, *DF* indicates the reflection axis corresponding to the incident axis, and the angle between *DF* and the incident optical axis is *α* = 2*θ*°. *F*_1_ and *F*_2_ represent the intersection points of the vertical line of the plane *P*_1_*OD* passing through *P*1 and the aperture. $A_{0}\left(x,0\right)$ denotes any point in the line segment where the light spot and the plane *P*_1_*OD* intersect. $A_{y}\left(x,y\right)\;$ represents any point in the spot. Accordingly, the distance between $A_{y}\left(x,y\right)\;$ and point *G* is $\sqrt{y^{2}{+\mathrm{AG}}^{2}}$, and $I_{A_{y}}$ represents the light intensity of this point.

Light continues to propagate as a conical beam when propagation from the reflection to the lens. Therefore, the cross-sectional graph intersecting the plane $A_{y}A_{0}G$ and the approximate conical beam form a complete circle. The light intensity distribution in the circle satisfies the Gaussian distribution. The standard deviation σ of the distribution remains the same, and *GL* indicates the radius of the circle. According to the Gaussian probability density function f(x)=12πσexp(−x22σ2), the central light intensity is inversely proportional to the standard deviation σ. If the central light intensity of the incident light is $I_{0}$ and the standard deviation is $\sigma_{\mathrm{in}}$, then the light intensity at position *G* is I0σinσAyA0G. Furthermore, because σin=r2, the light intensity at A0(x,0) can be calculated using Equation (1):

(1) $$I_{A_{0}}=I_{0}\frac{r}{GL}\exp\left(\frac{-{\left(AG\right)}^{2}}{2\times {\left(\frac{GL}{2}\right)}^{2}}\right),$$

where AG=(−x+f×tan2θ)×cos2θ).

Hence, the light intensity $I_{A_{y}}$ at any point $A_{y}\left(x,y\right)$ in the spot can be expressed as

(2) $$I_{A_{y}}=I_{0}\frac{r}{GL}\exp\left(\frac{-{\left(A_{y}G\right)}^{2}}{2\times {\left(\frac{GL}{2}\right)}^{2}}\right),$$

where $\{\begin{array}{c} A_{y}G=\sqrt{AG^{2}+{\left(A_{0}A_{y}\right)}^{2}}=\sqrt{{\left((-x+f\times \tan2\theta )\cos2\theta \right)}^{2}+y^{2}}\\ GL=(f/\cos2\theta -(-x+f\times \tan2\theta )\times \sin2\theta )\times r/f \end{array}$ When the measured surface is deflected in two dimensions, the processing method adopted herein is equivalent to 1D deflection, and Equation (2) is used to solve it. As displayed in Figure 3b, *P*_2_ indicates the reflected light corresponding to the light incident along the optical axis after the 2D deflection of the measured plane, which is tilted by 2*θ*_1_° and 2*θ*_2_° in the directions of $\vec{{\mathrm{OP}}_{\theta 1}}$ and $\vec{{\mathrm{OP}}_{\theta 2}}$, respectively. The rotation angle η is defined as

(3) $$\eta =\{\begin{array}{cc} -\arctan\frac{\tan2\theta_{2}}{\tan2\theta_{1}}, & 0<\theta_{1}<\frac{\pi }{2}\\ \pi -\arctan\frac{\tan2\theta_{2}}{\tan2\theta_{1}}, & -\frac{\pi }{2}<\theta_{1}<0\\ -\frac{\pi }{2}, & \theta_{1}=0\cap^{\;}0\leq \theta_{2}<\frac{\pi }{2}\\ \frac{\pi }{2}, & \theta_{1}=0\cap^{\;}-\frac{\pi }{2}<\theta_{2}<0 \end{array}.$$

Here, *P*_2_ can be regarded as *P*_1_ obtained using the coordinate rotation angle transformation, as expressed in Equation (4):

(4) $$\left[\begin{array}{l} x\\ y \end{array}\right]=\left[\begin{array}{cc} \cos\eta & -\sin\eta \\ \sin\eta & \cos\eta \end{array}\right]\left[\begin{array}{l} \tilde{x}\\ \tilde{y} \end{array}\right].$$

As shown in Figure 3b, *OP*_2_ = *OP*_1_ = $f\times \sqrt{\tan^{2}{\;2\theta }_{1}{+\tan}^{2}{\;2\theta }_{2}}$, and ${\tilde{A}}_{y}\left(x,y\right)$ and ${\tilde{A}}_{0}\left(x,0\right)$ correspond to $A_{y}\left(x,y\right)$ and $A_{0}\left(x,0\right)$, respectively. The one-dimensional equivalent tilt angle $\tilde{\theta }$ after the 2D tilt transformation in the rotating coordinate system can be obtained using Equation (5):

(5) $$\tilde{\theta }=\{\begin{array}{c} \frac{1}{2}\arctan\sqrt{\tan^{2}2\theta_{1}+\tan^{2}2\theta_{2}},\theta_{1}\geq 0\\ -\frac{1}{2}\arctan\sqrt{\tan^{2}2\theta_{1}+\tan^{2}2\theta_{2}},\theta_{1}<0 \end{array}.$$

We added the character “~” to the character mark of the equivalent 1D inclination to distinguish the 1D inclination and the equivalent 1D inclination obtained by rotating the spatial coordinates from the 2D inclination, which is the same as the 1D inclination mark in Figure 3a:

(6) $$I_{{\tilde{A}}_{y}}=I_{0}\frac{r}{\tilde{G}\tilde{G}}\exp\left(\frac{-{\left({\tilde{A}}_{y}\tilde{G}\right)}^{2}}{2{\left(\frac{\tilde{G}\tilde{L}}{2}\right)}^{2}}\right),$$

where $\{\begin{array}{c} {\tilde{A}}_{y}\tilde{G}=\sqrt{{\left((-(\cos\eta \times \tilde{x}-\sin\eta \times \tilde{y})+f\times \tan2\tilde{\theta })\cos2\tilde{\theta }\right)}^{2}+{\left(\sin\eta \times \tilde{x}+\cos\eta \times \tilde{y}\right)}^{2}}\\ \tilde{G}\tilde{L}=(f/\cos2\tilde{\theta }-(-(\cos\eta \times \tilde{x}-\sin\eta \times \tilde{y})+f\times \tan2\tilde{\theta })\times \sin2\tilde{\theta })\times r/f \end{array}$.

Although the surface of the measured object satisfies the law of reflection, an absolute, smooth outcome cannot be achieved when the confocal system measures the surface topology of an object. In other words, the tested surface cannot be completely smooth; it should have a certain degree of roughness. Moreover, owing to factors associated with other components in the optical path, such as processing and installation, the light spot returning to the cross-sectional position of the optical path does not yield a perfect Gaussian distribution, and it contains a small amount of streak-like structures, i.e., speckles [28]. A speckle exists in the experimental data as a common type of noise affecting the peak-extraction process and the experimental results. Moreover, the noise generated by the environment and the line scan CCD camera also affects the experimental results. Hence, the mathematical model should consider these problems and propose an appropriate approach to reduce its influence using advanced peak-extraction algorithmic processing.

**Figure 4:**
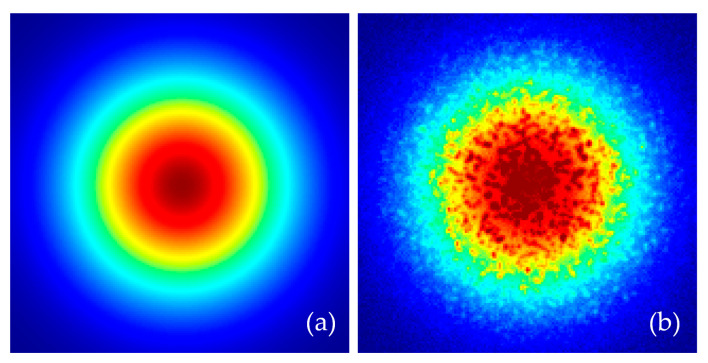
Simulation diagram of light-field distribution in front of a cylindrical lens. (**a**) Gaussian beam without noise; (**b**) consideration of speckle and environmental noise.

Consider that a speckle only exists in the experimental data as noise. This study approximated it as a normal speckle to simulate its statistical characteristics and randomly generated Ns speckles in the cross-section of the light cone to simplify the model. In this model, the central intensity of all speckles in the cross-section and the light intensity distribution of a single speckle follow a Gaussian distribution. Upon considering the effects of speckle and environmental noise, and in the case the measured surface was not tilted, the light field distribution of the return optical path section is illustrated in Figure 4.

The above model was established to predict the 2D response of the system at different surface inclinations. Unlike previous theoretical models that lacked features to process the 2D input intensity distribution and approximate the light spot to a perfect circle, this model facilitates a more accurate simulation of the system response to tilt and establishes a simulation model to verify the capability of the peak-extraction algorithm after considering the influences of laser speckle and environmental noise. Although this model proposed several assumptions and approximations, there are several aspects that have not been explored in this study because of practical limitations and will be explored in the future to simulate the real response of the system more accurately.

**Figure 5:**
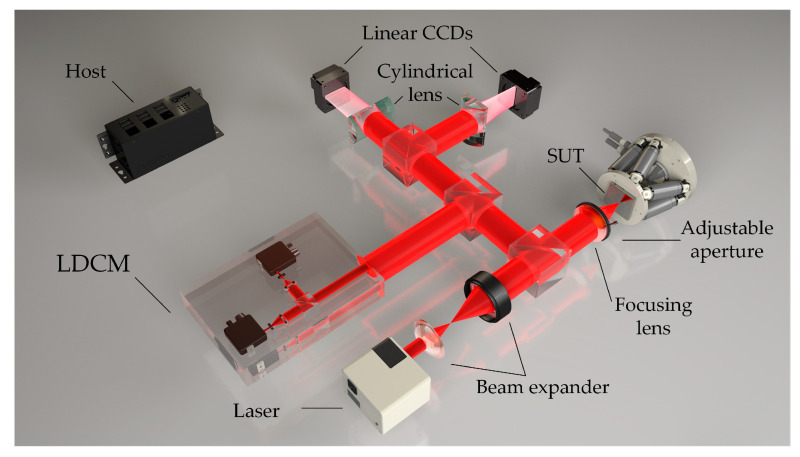
Schematic of a laser differential confocal microscope using the reported two measurement units whose spatial positions are orthogonal to each other. Each measuring unit comprises a cylindrical mirror and a linear charge-coupled device (CCD).

The system structure displayed in Figure 5 was designed to accurately measure the 2D angle deflection of the measured surface in the confocal system. Accordingly, two angle measurement units placed perpendicular to each other were added to the confocal system, and each measurement unit was composed of a cylindrical mirror and a linear CCD detector. The Gaussian beam from the laser converges at the objective of the microscope via a beam splitter and is focused on the surface of the measured object that was deflected in 2D. Once the diaphragm blocks the returning deflected beam, part of the light becomes a collimated beam after it passes through the microscope. After passing through the splitter, the collimated beam is split into two beams. One light beam enters the detection link of the CM or LDCM (the selected diagram depicts the LDCM, and details of the specific structure are not described here). The other beam passes through the beam splitter and enters the detection module composed of the cylindrical mirror and the linear array CCD, which are perpendicular to each other. The linear array CCD transmits the light intensity along with the pixel distribution data to the data acquisition board (DAQ) before sending it to the host computer. The peak-extraction algorithm computes the peak energy positions on the two linear array CCDs, and a stable mapping relationship between the peak position and the angle is obtained by fitting the polynomial surface function. Notably, the DAQ controls the timing of the system in a unified manner. When the objective lens is cyclically scanned, the detector of the differential confocal system or the confocal microscope system records real-time data, and the two linear array CCDs are orthogonal to each other. Data are recorded simultaneously, and the inclination angle is solved using advanced algorithms. The confocal system has a unique axial slicing ability, which enables it to accurately obtain the focal position and time to ensure the angle measurement satisfies the reflection law and ignores the problems caused by the defocused state. Specifically, the return beam is parallel to the proposed sensor, and the confocal structure ensures that the return beam is parallel. Thus, the confocal system is a prerequisite to using the sensor.

The combined structure of cylindrical mirror and linear array CCD replaces the area array CCD, and overcomes the difficulty associated with the low-response speed of the area array CCD, which cannot provide real-time measurements of the optical probe of the profiler across the same detection area. Meanwhile, all the pixels on the linear CCD can approximately guarantee the same photosensitive ability. Thus, the predicted and actual positions maintain a good linear relationship without the nonlinear error problem caused by the PSD owing to the larger deflection angle [25]. Unlike the system that uses a PSD structure, the proposed system structure supports the realization of advanced extraction algorithms and reduces the influence on the angular measurements as the reflectivity of the measured object and energy of the light source change. The SNR of the CCD improved owing to the good light-gathering ability of the cylindrical lens, while the intensities of the dark noise caused by CCD and the stray light caused by other directions in the environment were reduced.

Cylindrical lenses are extensively utilized in imaging and light source shaping, and the thickness of the light sheet can be achieved by changing their NA [29,30,31]. A cylindrical mirror can encode 1D information without affecting information in another dimension and can be used in conjunction with a line detector [32]. When the pixel of the linear array CCD pixel satisfies $d_{\mathrm{LCCD}}\text{ >> }2{.66\;\lambda f}_{cl}/d_{cl}$, it satisfies the requirements of this system. More specifically, the main part of the energy exceeding 93.8% of the total energy is received by the linear array CCD, where λ symbolizes the system wavelength, $f_{cl}$ represents the focal length of the cylindrical mirror, and $d_{cl}$ denotes the diameter of the cylindrical mirror.

To obtain the mathematical model of the light intensity-pixel distribution on the linear CCD, the function of the cylindrical mirror in the system is approximated as that of an ideal integrator. Accordingly, two assumptions are postulated as those postulated in the focusing of the microscope. First, the linear CCD is precisely placed on the focal plane of the cylindrical lens without offset. Second, when the SUT is not tilted, the center of the beam coincides with the position of the center pixel of the linear CCD. Accordingly, the intensity at any point on the CCD side is obtained by integrating the 2D inclined light spot model generated by the surface of Equation (6). The energy of any pixel on the CCD is obtained by using the pixel size as the integration area. The intensity of any point on the two line-array CCDs is expressed using Equation (7):

(7) $$\begin{array}{l} \{\begin{array}{c} I(\tilde{x})=\int_{-\sqrt{R^{2}-{\left(\tilde{x}\right)}^{2}}}^{\sqrt{R^{2}-{\left(\tilde{x}\right)}^{2}}}I_{0}\frac{r}{GL}\exp\left(\frac{-{\left({\tilde{A}}_{y}G\right)}^{2}}{2\times {\left(\frac{GL}{2}\right)}^{2}}\right)d(\tilde{y})\\ I(\tilde{y})=\int_{-\sqrt{R^{2}-{\left(\tilde{y}\right)}^{2}}}^{\sqrt{R^{2}-{\left(\tilde{y}\right)}^{2}}}I_{0}\frac{r}{GL}\exp\left(\frac{-{\left({\tilde{A}}_{y}G\right)}^{2}}{2\times {\left(\frac{GL}{2}\right)}^{2}}\right)d(\tilde{x}) \end{array} \end{array}$$

When the measured surface is tilted in one dimension, the light intensity distribution curves on the two mutually perpendicular CCDs are interdependent. Specifically, the energy distribution curves in the two linear array CCDs simultaneously change when the angle in the other dimension changes while the 1D inclination angle remains unchanged. Two specific distribution curves, namely, two specific peak pixel positions, correspond to a 2D tilt angle. Thus, the proposed measurement method must fit two 3D surfaces to represent the real-time 2D tilt of the measured surface. The two surfaces describe the corresponding relationship between the peak positions on the linear CCDs whose deflection angles are perpendicular to each other in space.

## 3. Modified Peak Extraction Strategy

When the confocal system is used to measure the surface topography of objects, the curve distribution of the light intensity at the linear CCD end changes according to the change in the number of pixels owing to the different reflectivity properties of the measured objects and the same tilt angle in the structure proposed herein, and a large float occurs. The advanced peak-extraction algorithm avoids measurement errors caused by changes in the intensity of the light source or unbalanced reflectivity of the measured object. Thus, the extraction precision, robustness, anti-noise capability, and speed of the peak–extraction algorithm directly affect the system precision and processing speed.

Common standard peak-wavelength extraction algorithms include maximum search, centroid algorithms, and model-based Gaussian fitting. The maximum search selects the discrete wavelength with the greatest intensity as the peak wavelength. It is relatively simple to implement the maximum search; however, the search results are rather noisy.

The centroid algorithm is fast and direct. However, as the surface inclination increases, the asymmetry of the peak curve on the linear CCD gradually increases. The centroid algorithm has large errors, and the limited sampling of pixels causes systematic errors [33]. Model-based fitting algorithms, such as polynomial and Gaussian algorithms, fit the recorded signals to polynomial and Gaussian functions and extract peaks as the results [34]. Despite having the highest extraction accuracy, it is difficult to use model-based fitting algorithms to satisfy the real-time performance requirements of the optical probe on the profiler owing to its computationally complex and time-consuming characteristics. Meanwhile, as the surface inclination angle of a certain dimension increases, the data starts showing obvious asymmetry. In a single dimension, the peak extraction error based on the model fitting algorithm slightly increases owing to the lack of data in a certain direction. The centroid extraction algorithm and Gaussian fitting are expressed using Equations (8) and (9):

(8) $$\mathrm{CA}:\;P_{c}=\frac{\sum_{i=1}^{n}P_{i}I_{i}}{\sum_{i=1}^{n}I_{i}},$$

(9) $$\mathrm{GFA}:\;\left|I_{i}-A\times \exp\left[-\frac{{\left(P_{G}-P_{i}\right)}^{2}}{2\times \sigma^{2}}\right]\right|\to \min$$

where $P_{c}\;$ represents the centroid position, *n* indicates the total number of samples, $P_{i}\;$ and $I_{i}\;$ represent the pixel data sequence position and the gray value in the linear CCD grayscale image, respectively, *A* represents the amplitude value of the Gaussian function, $P_{G}\;$ represents the sample expectation, and σ symbolizes the standard deviation. Thus, a peak-extraction algorithm that can consider both computational accuracy and computational efficiency is urgently required for the proposed system structure (shown in Figure 4).

As a clustering algorithm, the mean shift is extensively utilized in the fields of image processing and machine vision [35]. Its core idea involves searching for peaks along the upward direction of the probability density gradient—specifically toward a denser position. Peak finding based on the mean–shift vector is an iterative process, while the mean–shift vector always points in the direction of increasing probability density. The kernel density estimation achieves a maximum value when the mean–shift vector is zero. The algorithm was first proposed in 1975 by Fukunage [36]. It was subsequently improved and expanded by Cheng [37], who also defined a kernel function. When the distance between the reference and the sample points is different, the contribution of the sample point to the mean–shift vector is also different. To specify the significance of different sampling points in the process of obtaining the mean–shift vector, the weight coefficient is set in such a way that it is not completely consistent. For instance, when affected by noise, signal points with high-SNR values can be set to have larger weights. Lu [38] transformed the mean–shift theory of the spectral confocal displacement sensor (typically expressed in 2D form) into 1D form in the process of signal wavelength peak extraction. Accordingly, it exhibited comprehensive performance in terms of running speed and extraction accuracy. Additionally, the energy values corresponding to different wavelengths were regarded as density values to determine the wavelength position corresponding to the measured object, and the Gaussian kernel function was also utilized, as shown in Equation (10):

(10) $$G(\lambda )=\frac{1}{\sqrt{2\pi }}\exp\left(-\frac{1}{2}\lambda^{2}\right),$$

where λ symbolizes the wavelength sequence in the spectral confocal peak extraction system. In the process of spectral, confocal peak extraction, the mean–shift algorithm exhibits robustness and increased extraction speed characteristics throughout the entire domain. Such attributes primarily depend on the size and distribution of the data, namely, the distribution curve of the spectral confocal displacement sensor peak extraction system contains minimal data, a narrow half-peak width, obvious peaks, and a very high-SNR that is insensitive to environmental factors and speckle noise. Moreover, for an extremely small asymmetry degree in the distribution curve of the peak-value-extraction system of the spectral confocal displacement sensor, the change of the kernel function does not have a considerable impact on the extraction process where the kernel radius of the system is fixed. Nevertheless, the proposed system needs to choose a larger kernel radius when using mean–shift-based peak extraction algorithm with Gaussian kernel function (MSG) to deal with the light intensity pixel distribution curve with higher degrees of asymmetry, lower SNRs, wider half-peak widths, and larger data volumes. Consequently, the computational efficiency and extraction accuracy of MSG deteriorate. Meanwhile, the kernel function selection also determines the accuracy, robustness, and processing efficiency of the extraction algorithm.

The Epanechnikov kernel function (EK) is an excellent kernel function owing to its convenient calculation and centralized distribution [39]. As a kernel function, EK has achieved decent results in many kernel density estimation applications. Although EK has the same density estimation and data smoothing ability as the Gaussian kernel function, it has higher computing power than the Gaussian kernel function. First, the Epanechnikov kernel function does not need to call the exponential function. Second, all data values are not used for calculating the current iteration process [40]. Thus, the use of EK also reduces the effect of external noise on peak extraction beyond the non-kernel radius. Essentially, the kernel function is a weighted operation on the data. On the basis of improving the computing power of the system, the kernel function can cope with the noise problem with large randomness near the peak value to further improve the extraction accuracy and the robustness of the system. Therefore, a modified kernel function that combines EK and the rectangular kernel function is proposed:

(11) $$K(x)=\{\begin{array}{c} \frac{3}{4-4m^{3}}\left(1-x^{2}\right),\;m\leq |x|\leq 1\\ \frac{3}{4-4m^{3}}\left(1-m^{2}\right),\;\mathrm{otherwise} \end{array},$$

where *K*(*x*) represents the novel unit kernel function, and m indicates a variable parameter. 

Assume that the line array CCD pixel sequence is $\vec{P}$, and $\vec{I}$ indicates the corresponding current light intensity sequence, *w*(*x*) represents the weight function, μ symbolizes the power of the weight function, and *h* indicates the kernel radius. In this study, when the inclination angle is zero, the half-peak width of the distribution curve indicates the kernel radius of the kernel function. $P_{\mathrm{cur}}$ indicates the data point position of the current position kernel radius, and ε symbolizes the given (allowable) error. The overall algorithm is described in Algorithm 1.
**Algorithm 1.** Mean-shift based extraction with the novel kernel function**Input:** Pixel data sequence:$\vec{I}=\left[I_{1}{,I}_{2},\cdots {,I}_{N}\right],$ kernel radius: *h* Weight function power: μ, Iterative convergence threshold: ε **Output:** peak position of $\vec{I}$: $P_{\mathrm{cur}}\left(n\right)$ 1: Initialize $P_{\mathrm{cur}}\left(0\right)\;\leftarrow$ position of the maximum value in sequence $\vec{I}$
, *n* ← 0 2: Repeat 3:    n←n+1 4:   $P_{cur}(n)\leftarrow \frac{\sum_{i=P_{cur}(n-1)-h}^{P_{cur}(n-1)+h}i\times K\left(\frac{i-P_{cur}(n-1)}{h}\right)\times w\left(I_{i}\right)}{\sum_{i=P_{cur}(n-1)-h}^{P_{cur}(n-1)+h}K\left(\frac{i-P_{cur}(n-1)}{h}\right)\times w\left(I_{i}\right)}$, where *w*($I_{i}$) = ${{(I}_{i})}^{\mu }$ 5: Until convergence: $\left|P_{\mathrm{cur}}(n)-P_{\mathrm{cur}}(n-1)\right|<\varepsilon$

In previous studies [41], a low-order polynomial was directly used for fitting the polynomial surface of discrete points in space. It is suitable for the small-angle system calibration process because the number of discrete spatial points is small, and the low-order polynomial can be well-fitted. However, the fitting accuracy does not reach the ideal state for a large angle range. If the order of the fitting polynomial curve is increased, there are ill-conditioned matrices and overfitting. In addition, it is highly sensitive to dead pixels, which deteriorates the accuracy of the system. Thus, a partition-fitting algorithm suitable to enhance measurement accuracy is proposed.

**Figure 6:**
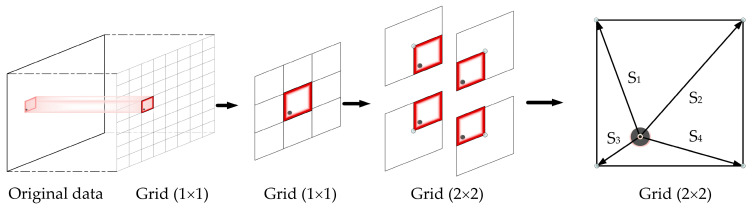
Schematic of the partition-fitting strategy.

As depicted in Figure 6, once the peak extraction algorithm obtains the peak coordinates, the 2D plane formed by the two linear CCD pixel sequences is divided by the step size D_C_. Therefore, the present peak coordinates are used to determine the “best partition” based on the closest distance principle. The specific implementation steps are outlined in Algorithm 2.
**Algorithm 2.** Partition-fitting strategy**Input:** Horizontally placed CCD train set: {*h*_1_, *h*_2_, …, *h_k_*}    Vertically placed CCD train set: {$v_{1}{,v}_{2},\ldots {,v}_{k}$},    Size of fitting matrix: {*m*, *n*} **Output:** Fitting blocks: $\left\{\begin{array}{ccc} f_{1,1} & \cdots & f_{1,n}\\ \vdots & \ddots & \vdots \\ f_{m,1} & \cdots & f_{m,n} \end{array}\right\}$ 1: Obtain the peak position of train set by using Algorithm 1 2: Calculate the maximum and minimum values of peak positions in the train set: *H*_max_, *H*_min_, *V*_max_, and *V*_min_ 3: for *i* = 1:*m* do for *j* = 1:*n* do Center of *f_i_*_,*j*_: $C_{i,j}\leftarrow \left(H_{\min}+(i-1)\times \frac{H_{\max}-H_{\min}}{m},V_{\min}+(j-1)\times \frac{V_{\max}-V_{\min}}{n}\right)$ Initialize: *k*←1 Repeat Find the train set centered on *C_i_*_,*j*_ within $k\;\times \;\frac{H_{\max}-H_{\min}}{m}$ horizontally and $k\;\times \;\frac{V_{\max}-V_{\min}}{n}$ vertically: *f_i_*_,*j*_trainset_ *k* ← *k* + 1     until the number of train set in *f_i_*_,*j*_trainset_ is greater than the minimum value     Use the data in *f_i_*_,*j*_trainset_ to fit *f_i_*_,*j*_   end for  end for 


In the calibration experiment, the calibration surface is obtained using the training dataset. By using the test set or the actual operating condition, the method to predict the current angle once the system returns the peak positions of the two linear CCDs is outlined in Algorithm 3.
**Algorithm 3.** Use of the partition-fitting strategy to predict the tilt angle**Input:** Horizontally placed CCD experimental data: h,     Vertically placed CCD experimental data: v,    Fitting partitions: $\left\{\begin{array}{ccc} f_{1,1} & \cdots & f_{1,n}\\ \vdots & \ddots & \vdots \\ f_{m,1} & \cdots & f_{m,n} \end{array}\right\}$    Center of fitting partitions: $\left\{\begin{array}{ccc} C_{1,1} & \cdots & C_{1,n}\\ \vdots & \ddots & \vdots \\ C_{m,1} & \cdots & C_{m,n} \end{array}\right\}$ **Output:** Predicted tilt angle: {anglel, angle2} 1: Obtain the peak position of experimental data using Algorithm 1: [peak_h, peak_v] 2: Through the center of the fitting partitions, find the fitting partition closest to the peak of the experimental data: C_*p*,*q*_, where *p*
∈ [0, *m*], and *g*
∈ [0, *n*]


## 4. Validation of Simulation Performance

**Figure 7:**
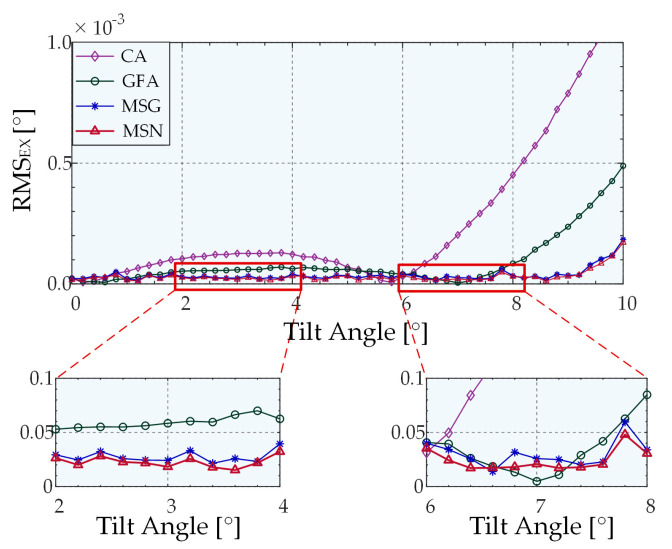
Peak-extraction errors of different algorithms based on simulations.

When the measured object is tilted in any 2D configuration, the light intensity distribution curve on two-line array CCDs that are perpendicular to each other can be simulated using Equation (7). Accordingly, the speckle model and the noise effect are added to the data to simulate the actual light intensity distribution on the linear CCD in the physical experiment environment, and the optimal peak extraction algorithm for the system is obtained by comparison. The system parameters were set in the simulation model as follows: the radius of the incident Gaussian light source is 2 mm, the radius of the diaphragm placed on the focusing surface of the microscope objective is 4 mm, the focal length of the microscope objective is 9 mm, the number of pixels of the linear CCD is 2048, and the pixel size is 7.04 μm × 7.04 μm. Subsequently, the simulation parameters were adjusted based on numerous simulation experiments. During the process, the simulated state of the system is closer to the actual result when adding Gaussian noise, which sets the SNR and the speckle number to 30 and 3000, respectively. The parameters of the algorithm were set as follows: the kernel radius is half of the half-peak width when the system does not deflect. The simulation results reveal that the power of the weight function *μ* is 1.4 under the current system parameters. The tunable parameter m in the kernel function was set to 0.4, which maximized the extraction accuracy. The simulation model proposed in this study has centrosymmetric properties on the 2D plane. The purpose of the simulation experiment is to compare the peak extraction capabilities of different algorithms. Thus, in the simulation experiment, only the peak extraction results on the linear array CCD were simulated for 1D deflection. We performed 500 trails at each given angle to ensure high confidence. The simulations were performed in the following manner: for each given angle, the pixel size was set to 0.1 as the sampling interval to generate simulation noise-free data, and the maximum value method was adopted to obtain the nominal value. We added the speckle model and the noise signal (SNR = 30) to obtain the light-field distribution in front of the cylindrical lens, accumulated separately, and the size of a single pixel was used as the integration interval to obtain each distribution curve of light intensity with pixels on a linear array CCD. The extracted peak value was obtained after using a variety of peak extraction algorithms, and the absolute value of the difference between the nominal value and the extracted peak value was used as the extraction error. The mean value of 500 groups of extraction errors was used as the given value extraction angle error. Finally, the extraction error curve with the angle was plotted, as shown in Figure 7.

Figure 7 illustrates the peak-extraction error of the four algorithms as a function of the tilt angle when only 1D tilt occurs. Fifty sets of 30 dB random Gaussian noise and speckle model noise were added to each fixed angle noise-free model, and the average value was calculated as the angle peak extraction error. As shown, at the current SNR, the Gaussian fitting and centroid extraction algorithms exhibit strong extraction abilities when there is no tilt or a slight tilt; these abilities are slightly higher than that of MSN. However, as the tilt angle increased, the errors of the centroid algorithm and the Gaussian algorithm were much larger than those of the two algorithms based on the mean–shift algorithm. Meanwhile, the extraction error is consistently smaller than the MSG when using the MSN algorithm, and the mean errors were 0.0438° and 0.0403°, respectively, which proves that the extraction accuracy of the MSN algorithm was higher compared to the accuracy of other algorithms. The variances of the four algorithms were all within the range allowed by the system (<0.0008). Except for the centroid algorithm, there is no phenomenon associated with the increase as a function of the angle. The variance of Gaussian fitting was much smaller than that of the other three algorithms, among which the average variance of MSN was 41.8% less than that of MSG. These outcomes prove that the algorithm has better robustness owing to the change of the kernel function.

**Figure 8:**
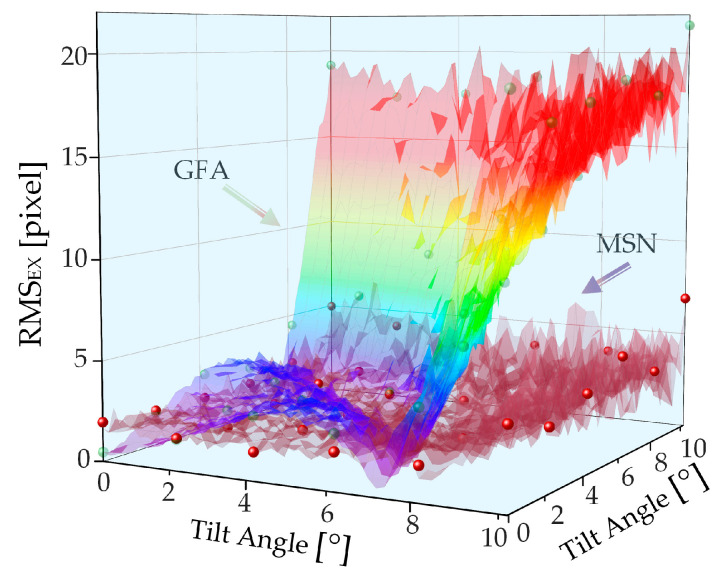
Simulation results of the root-mean-square values of the peak extraction errors of two CCDs when exposed to 2D tilt.

Upon verifying the ability to extract peaks using 1D tilt, we concluded that, when a 2D tilt occurs, the variation trends of the root-mean-square of the extraction error is represented as a function of the angle in the two dimensions of the Gaussian fitting and the MSN algorithms, as displayed in Figure 8. When a 2D tilt angle occurs, the trend of the error as a function of the angle is consistent with that of the 1D tilt.

**Figure 9:**
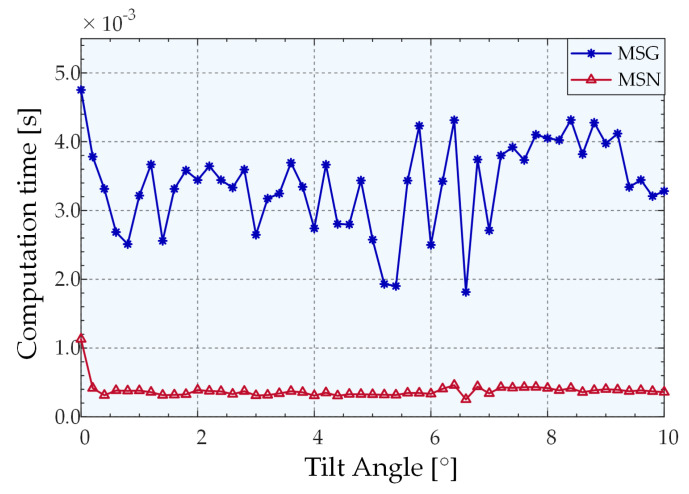
Comparison of the average processing times of MSG and MSN.

To evaluate the computational efficiency of different algorithms, we performed 1000 trails at each angle to avoid randomness and make the simulations reliable. A 2.9 GHz AMD R7-4800 h central processing unit (CPU) was used to compute the average time for extracting angles using various algorithms. The comparison of processing times of the MSG and MSN algorithms are presented in Figure 9. Following the change of the new kernel function, the speed exhibited a considerable improvement, and the processing speed curve of the MSN algorithm changes more smoothly. The average extraction times for CA, GFA, MSG, and MSN were 8.9992 × 10^−6^ s, 0.0535 s, 0.0064 s, and 0.0004 s, respectively. The processing speed of MSN was 18.15 times higher than that of MSG, and 152.3 times higher than that of Gaussian fitting. MSN can satisfy the speed requirements of the measurement system for the peak extraction algorithm.

## 5. Experiment and Discussion

**Figure 10:**
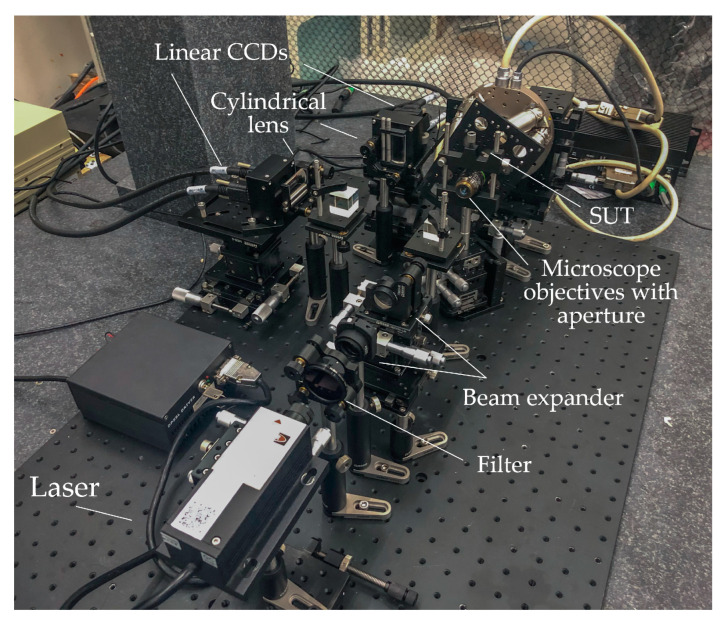
Experimental setup for the measurement system proposed in this study.

The experimental devices are displayed in Figure 10. A 532 nm laser with an output power of 10 mW (model, Coherent, Changchun New Industries Optoelectronics, Changchun, China) was used for light illumination. It was expanded using a pair of lenses (AC080-010-Af = 10 mm and AC254-040-A-MLf = 40 mm) after passing through a filter (1000×, EX-Color ND1000, NiSi, Zhuhai, China). The light source exhibited good Gaussian characteristics, and e^−2^-diameter of the beam at the waist was 1 mm. Once the parallel light passed through a beam splitter and a microscope objective lens (LMPLFLN 20×, NA = 0.4, *f* = 9 mm, Olympus, Tokyo, Japan), it was focused on a six-degree-of-freedom ultra-high-precision stage (H-811.I2, ±10, Power Integrations, San Jose, CA, USA) on the plane mirror (GMH-11, HYGX, Guangzhou, China). The reflected beam became parallel after the light passed through a customized diaphragm (d = 8 mm, ±0.02 mm) and a microscope objective. After passing through the beam splitter, the light beams entered a pair of cylindrical mirrors (GLH15-20 × 10-010-VIS, *f* = 10 mm, HYGX, Guangzhou, China), whose spatial positions are orthogonal to each other. Finally, the light beams entered a line that was also an orthogonal array CCD (LA-GM-02K08A, 2048 × 7.04 μm, Teledyne DALSA, Waterloo, ON, Canada). The pixel size of the camera satisfies the requirements of the expression dLCCD>2.66λfcldcl, and the line array CCD collects multiple sets of current data in real time. In this study, 50 sets of real-time data were collected, and mean filtering was performed to minimize accidental effects. Additionally, the ultrahigh-precision motion platform used in the laboratory had a minimum motion increment of 2.5 μrad for each rotation axis, and repeatability of ±2 μrad, which satisfies the required accuracy. The calibration experiments and random angle test experiments can be performed to verify system accuracy and algorithmic validity. For the convenience of expression, the rotation dimensions of the rotating platform used in the physical experiments were defined as *R*_1_ and *R*_2,_ respectively, and planar coordinates were used to represent the 2D inclination angle.

**Figure 11:**
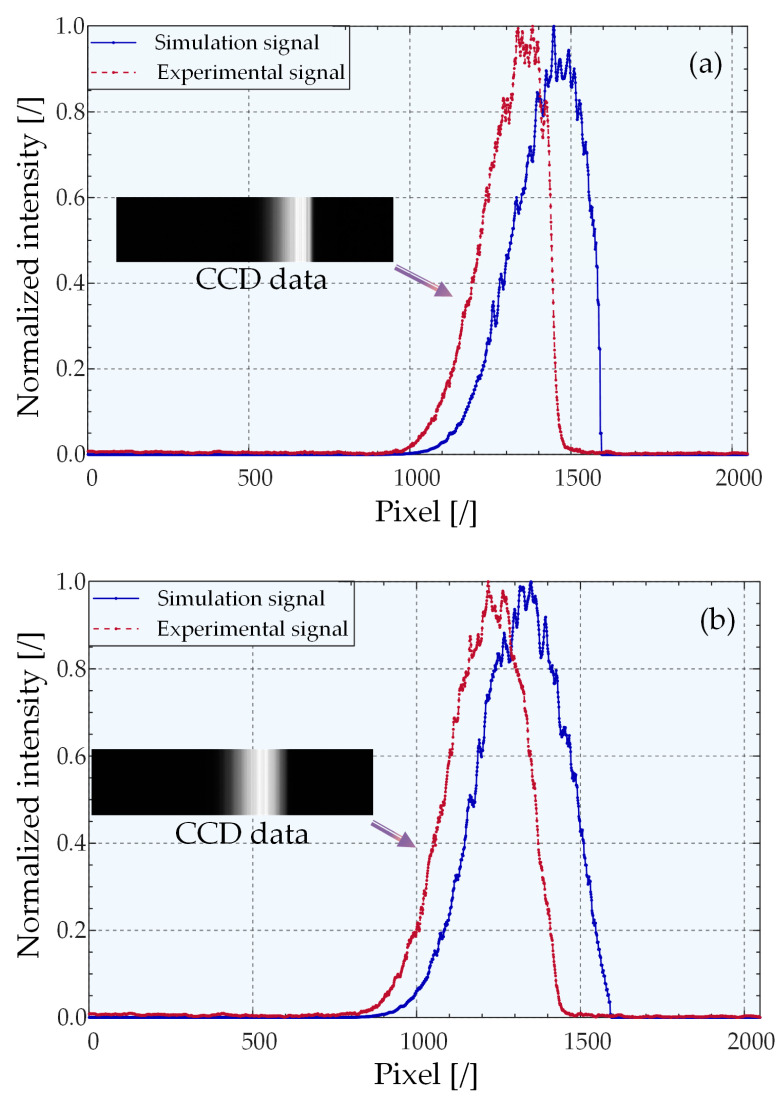
Simulation results and experimental data of light intensity-pixel distribution on a linear CCD. (**a**) (2°, 10°) on CCD1 and (**b**) (7°, 5°) on CCD2.

The data (2°, 10°) of linear CCD1 and linear CCD2 (7°, 5°) data were normalized and compared with the simulation model. The comparison outcomes are displayed in Figure 11. Moreover, a screenshot of the software integrated with the line array CCD is also provided.

There are differences between the simulation results and the physical experimental results. First, a difference exists in the width at half maximum. Second, the distribution positions cannot overlap. Following normalization, the same energy intensity has a fixed pixel position difference.

There is a dislocation in the overall distribution of the simulation and experimental results because the simulation experiment assumed that the position of the central pixel of the line array CCD was on the optical axis. In physics experiments, this cannot be achieved. However, misalignment does not induce any precision changes. The reasons for the differences in the width at half maximum may include the following: first, when the linear CCD is placed in the physical experimental setup, there will be an inevitable offset or tilt during the adjustment process, and there will be a linear image on the focal plane of the cylindrical mirror. It is impossible for the linear image on the focal plane of the cylindrical mirror to completely enter the linear array CCD, which makes the half-peak width narrower; second, the cross-section of the beam is not a perfect circle owing to the properties of the light source and the offset of the placement of other components in the optical path, which also induces changes in the distribution. Finally, the simulation model simulates the real system response. However, deficiencies need to be corrected in future studies.

Nevertheless, by comparing the experimental results with those of the simulation model, we cannot conclude that the model had the same distribution trend as the physical experimental data. It was proven that the simulation model could predict and simulate the light intensity distribution law on the linear array CCD when 2D tilts occurred. Meanwhile, the model verified the extraction accuracy, robustness, and speed responses of different peak extraction algorithms of the system.

In the calibration experiment, the measurement range was set from −10°–+10° in both dimensions, and the data were collected in increments of 0.1° for each rotational dimension to construct the fitted training dataset. Owing to the travel limitation of the rotating platform, it was impossible to simultaneously rotate the 2D data. In other words, the intention was to obtain a range of −10°–+10°. However, owing to limitations of the physical experimental device, the data acquisition process was affected, which in turn, affected the accuracy. More specifically, when one dimension reaches a large angle (greater than 9°), the angle of the other dimension can only reach a maximum of 7°. In total, we collected 34,936 datasets. Additionally, we randomly generated 8500 groups of test angles as the test set using the host computer software. To verify the accuracy of the system and ensure the randomness of the test, the minimum data interval of the test angle was set to 0.01°.

**Figure 12:**
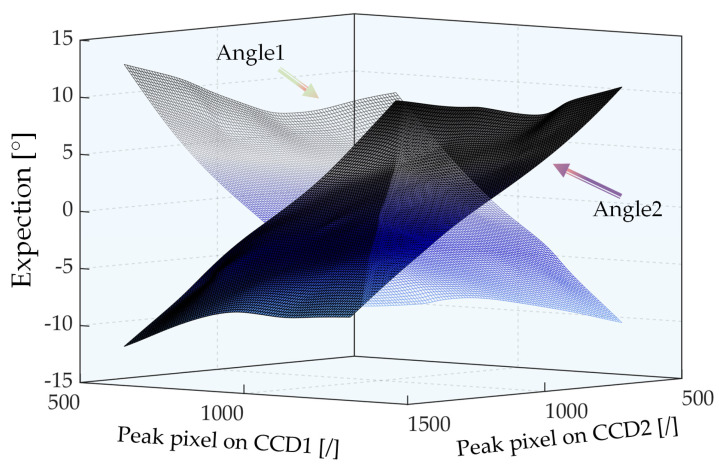
Fitted surface results for calibration experiments.

Initially, two calibration surfaces perpendicular to each other in space, as shown in Figure 12, were obtained based on a fifth-order polynomial surface fitting by using the advanced peak-extraction algorithm on the training dataset. The test dataset in step 2 was set to perform the angle predicting capability of the system measurements and algorithm comparison verification. When the two-line array CCDs were submitted to the system for the two peak pixel positions, two calibration surfaces were substituted to predict the current 2D tilt angle. The algorithm parameters in the physical experiment were set based on those in the simulation experiment.

**Figure 13:**
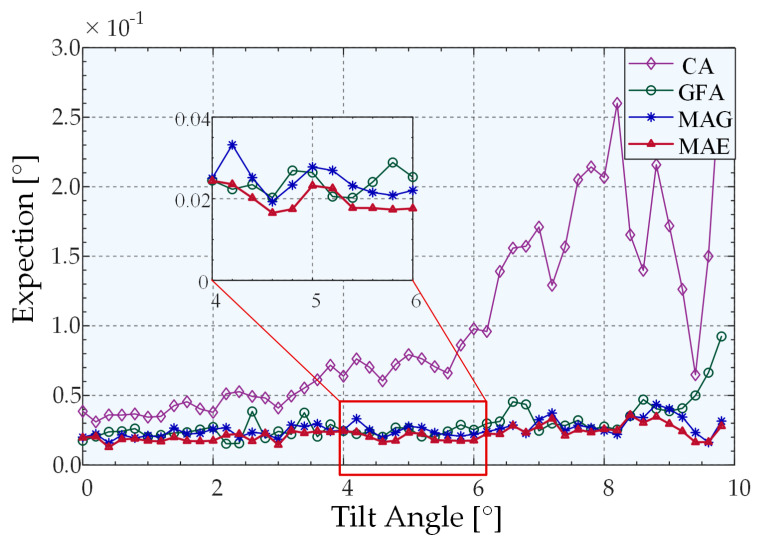
Angular prediction errors of different algorithms.

**Figure 14:**
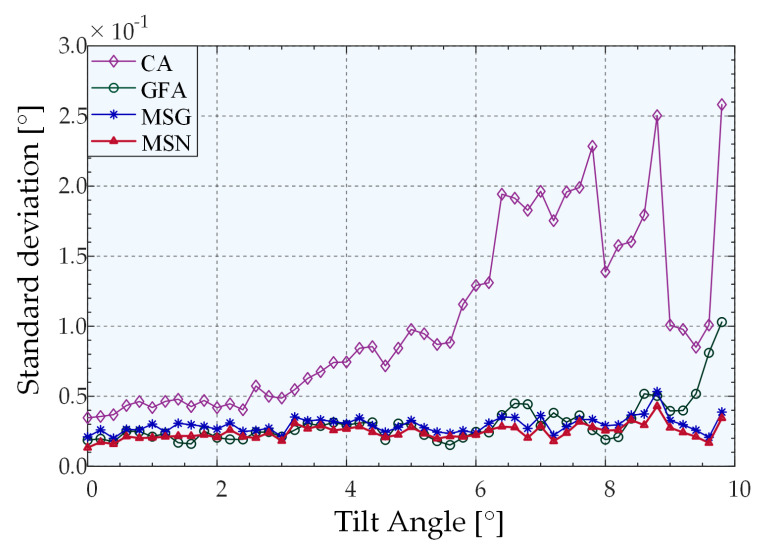
Angular prediction standard deviations of different algorithms.

Figure 13 and Figure 14 present the prediction error and standard deviation curves as a function of angle for different algorithms. The horizontal axis represents the tilt angle in the range of 0°–10° in dimension *R*_1_, and the sample selection interval is 0.2°. The vertical axis represents the average value of the angle prediction error obtained by calculating the average value of the angle prediction error of the current tilt angle of the *R*_1_ dimension in the test dataset, and all the angles of the *R*_2_ dimension are within its ±0.1° range. Based on these reasons, we processed and plotted the data. First, the prediction error and standard deviation of the experimental results on the 2D inclined plane adhere to the principle of centrosymmetry, and the superiority of the algorithm can be compared even when displaying the range of 0°–10°. Second, 3D data can be converted into 2D curves, which is convenient as this allows us to demonstrate that the change in the measurement angle affected the algorithm.

The predicted angle error and standard deviation exhibit an identical trend. More specifically, the predicted angle error and standard deviation of the centroid algorithm exhibit an apparent increasing trend as the angle increases. By contrast, the Gaussian fitting algorithm has a more stable distribution than the centroid algorithm. However, a clear upward trend exists after the angle reaches 9°. The two peak-extraction algorithms based on mean–shift theory exhibit a stable overall distribution. Furthermore, both the prediction error and standard deviation of the MSN algorithm are smaller than that of the MSG algorithm, thus indicating that the MSN algorithm has higher accuracy than the other three algorithms. Within the measurement range (*R*_1_,*R*_2_ ∈ [−10°, 10°]), the average prediction errors of the results obtained by using MSN, MSG, GFA, and CA peak-extraction algorithms combined with quintic polynomial surface fitting were 0.0220°, 0.0257°, 0.0299°, and 0.0986°, respectively. The average standard deviations were 0.0242°, 0.0294°, 0.0306°, and 0.1040°, respectively.

**Figure 15:**
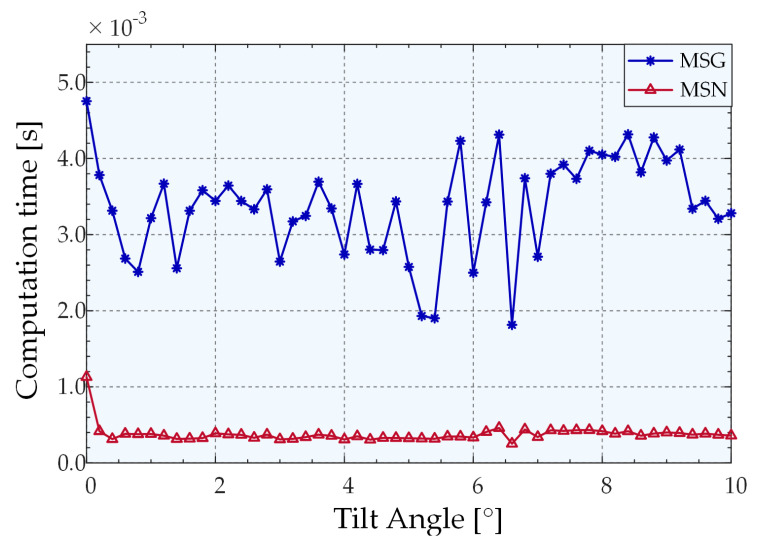
Extraction times of different algorithms.

We estimated the statistics on the average computational efficiency of different algorithms. As depicted in Figure 15, MSN exhibits a greater improvement than MSG in terms of the processing speed. The average extraction times of MSN, MSG, GFA, and CA in the overall measurement range were 0.000378783 s, 0.003380013 s, 0.067486125 s, and 1.22931 × 10^−5^ s, respectively. Evidently, the processing speed of the MSN algorithm is 8.923 times higher than that of the MSG algorithm and 178.166 times higher than that of the Gaussian fitting.

**Figure 16:**
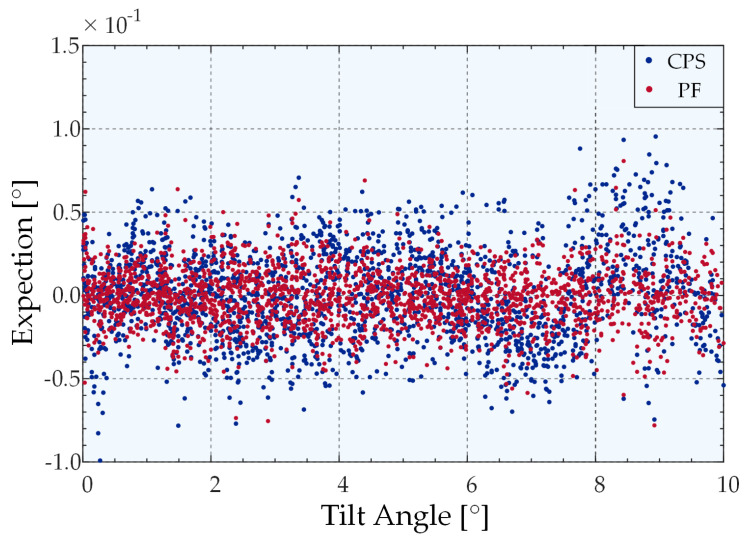
Angle prediction errors for different fitting strategies.

Following the use of the advanced peak extraction algorithm to accurately obtain the peak position, it is crucial to identify ways to select the appropriate fitting algorithm for the calibration surface to ensure the accuracy of the predicted angle. To clearly show the effectiveness of the proposed new partition fitting strategy, we compared the error performance associated with the use of the partition fitting strategy (PF) and classical polynomial surface fitting (CPS) subject to the premise of using the MSN peak extraction algorithm, as shown in Figure 16. The horizontal axis represents all the tilt angles in the range of 0°–10° in the *R*_1_ dimension in the test dataset. The ordinate represents the angle prediction error, where only 1D tilt occurs in the *R*_1_ dimension, and the projection of the *R*_1_ dimension from the angle error, where the two dimensions of *R*_1_ and *R*_2_ are simultaneously deflected. Figure 16 illustrates that the angle prediction error of the new partition prediction fitting strategy is significantly smaller than that of the fifth-degree polynomial surface fitting of the overall calibration data based on the assumption that the same peak-extraction algorithm was used. The average prediction errors of CPS and PF were 0.021495139° and 0.013430807°, respectively. Accordingly, it can be concluded that the accuracy of the system can be significantly enhanced by improving the prediction accuracy of the partition prediction fitting strategy by 37.53%.

**Figure 17:**
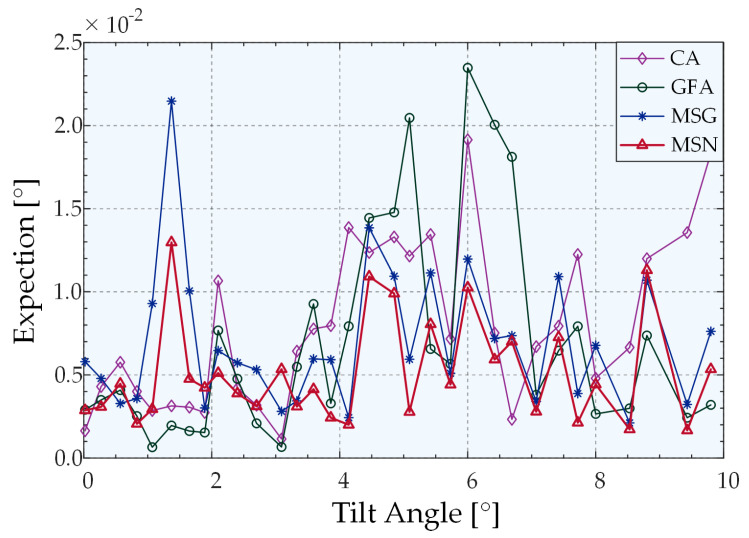
Algorithmic extraction performance with large energy fluctuations.

In actual operating conditions, the objects detected by the CM system are not necessarily all ideal test objects with constant reflectivity. If the reflectivity is different, the light-intensity–pixel distribution curve on the line array CCD changes. Although the pixel position of the peak remains unchanged, the change in the distribution affects the result of the peak extraction algorithm. Thus, the robustness of the peak extraction algorithm and the accuracy of peak finding are crucial. Therefore, we performed an alternative experiment. Specifically, we employed a laser with a large energy fluctuation that changed the light intensity distribution on the linear CCD to simulate the effect of the change in the reflectivity of the surface to be measured by the peak-extraction algorithm. The laser energy fluctuation was approximately ±15% of the nominal value. The scan was repeated thrice, and the average value of the prediction error was calculated. Figure 17 presents the results that verify the fact that the MSN peak-extraction algorithm is less affected by the change in the distribution of light intensity pixels. The high-precision displacement stage selected for the experiment ensured the positional accuracy of the repeated scanning experiments.

As listed in Table 1, different extraction algorithms can achieve an overall angle prediction accuracy within the overall measurement range for the same partition fitting strategy. Among them, the prediction accuracy of the MSN algorithm reaches 0.0134° (0.067% F.S). Analysis indicates that, although the measurement accuracy in the two dimensions maintained a uniform trend, there were similar differences among the algorithms with regard to the error. First, we used a beam quality analyzer to obtain a beam spot that presented an elliptical distribution after beam expansion. Second, during the experiment, the alignment error, including the alignment error of the cylindrical mirror and the linear CCD, could not be perfectly placed on the focal position of the cylindrical mirror. Third, the system used the Power Integrations company’s six-degree-of-freedom platform to conduct a K9 plane mirror as the measured object to collect training data. The motion range of the motion platform was asymmetric in the two axes, which resulted in different training data in the two axes. Consequently, the asymmetry in measurement accuracy was also affected. Finally, the motion of the two dimensions of the motion platform could not be completely coincident with the linear array CCD that presented an orthogonal relationship in spatial position, which resulted in the uneven density of the fitting data that ultimately resulted in different accuracy outcomes.

The accuracy of the measurement system proposed in this study was affected by the following: whether the distribution characteristics of the light source satisfied the Gaussian distribution and the stability of the light source, condition of the pixel size and resolution of the line CCD, selection of system components, including the numerical aperture of the light source radius microscope and parameter selection of the algorithm. The main application challenge of the sensor proposed in this paper is the fluctuation of light source and reflectance. In this study, the MSN algorithm is employed to suppress the fluctuation of light source and reflectance to the maximum extent; however, the effect is limited, so stable light source and the measured object with no great change of reflectance are required. The system has high requirements for the installation and adjustment accuracy, particularly the position of the linear CCD, which directly affects the measurement accuracy of the system. Correctly adjusting the radius of curvature of cylindrical mirrors can effectively balance the requirements of precision and assembly precision. The selection of curvature radius does not affect the range of angle measurement; however, it will significantly affect the resolution and accuracy. Theoretically, a smaller radius of curvature leads to better resolution and accuracy. However, in practice, the installation precision will significantly impact the accuracy of the system. If the curvature radius is too small, it results in a large error tolerance rate of installation and adjustment. Once it defocuses or tilts, the linear array CCD cannot accurately obtain all optical path information.Therefore, there is a trade-off between the radius of curvature and the capacity to install and adjust.

Unlike previous studies [23], the sensor proposed in this study realizes accurate real-time measurement of the two-dimensional inclination angle of the confocal system. Besides expanding the measurement range, the proposed sensor also improves the measurement accuracy [26]. Moreover, the use of the high-resolution linear array CCD simultaneously satisfies the requirements of response speed and accuracy of the system, indicating the applicability of the excellent peak extraction algorithm to two-dimensional detection for improving the detection accuracy of the sensor and system robustness, which were difficult to achieve in previous studies [19].

## 6. Conclusions

A precise, 2D tilt measurement sensor based on the double-cylindrical mirror structure for confocal microscopy systems was proposed in this study. The proposed sensor enables the confocal system to simultaneously measure the spatial position and tilt angle of the surface. Additionally, precise measurements of the inclination of the measured surface reduced the error in positional measurement caused by inclination and allowed dynamic optimization of the sampling strategy during 3D reconstruction. The efficient use of this sensor relied on the excellent ability of the confocal microscopy system to determine the focal plane as it allowed the angle measurement system to avoid effects of defocusing errors. 

The proposed angle measurement sensor has the characteristics of precision and high efficiency. The use of the cylindrical mirror combined with the linear CCD significantly improved the response speed so that the angle measurement could satisfy the speed required for position measurements. Considering the double-cylindrical mirror structure, a physical model that considered speckle and environmental noise was developed, and the system response was simulated. Moreover, a peak-extraction algorithm based on the new mean–shift kernel function was proposed to address existing problems in the system, such as extraction accuracy, extraction speed, and fitting error. It balanced the requirements of accuracy and speed. The average processing speed of MSN was 7.923 times higher than that of MSG and 177.166 times higher than that of traditional GFA. Furthermore, a novel partition-fitting strategy was proposed, which improved the prediction accuracy by 37.53%. In calibration experiments, it was determined that the light source had a wavelength of 532 nm. Each dimension ranged from −10°–+10°, the average predicted angle errors of the sensor’s Peak_1_-Peak_2_-Angle_1,2_ were 0.0134° (0.067% F.S) and 0.0142° (0.071% F.S), respectively, and the standard deviations were 0.0111° and 0.0119°, respectively. The measurement accuracy of the 2D angle can be further optimized based on system design optimization based on the actual applicational requirements. The proposed sensor enables the traditional confocal microscope optical probe to obtain accurate and simultaneous estimates of the 2D inclination angle of the surface and the spatial position coordinates of the measured surface.

## Figures and Tables

**Table 1:** Average prediction errors of different algorithms in the *R*_1_ and *R*_2_ dimensions based on the use of the novel partition prediction fitting strategy.

Algorithms	Error in *R*_1_ Dimension	Error in *R*_2_ Dimension
MSN	0.0134°	0.0142°
MSG	0.0156°	0.0169°
GFA	0.0153°	0.0165°
CA	0.0270°	0.0591°

## Data Availability

Not applicable.
